# Synthesis and Antiproliferative Activities of 5-Azacytidine Analogues in Human Leukemia Cells 

**DOI:** 10.3390/molecules13071487

**Published:** 2008-07-23

**Authors:** Gang Guo, Gang Li, Dan Liu, Qian-jiao Yang, Yu Liu, Yong-kui Jing, Lin-xiang Zhao

**Affiliations:** 1 Shenyang Pharmaceutical University, Shen yang, 110016, P. R. China; E-mails: guogang19761126@sina.com(Guo); ligang2001_21@yahoo.com.cn (Li); sammyld@163.com(Liu); yangqianjiao102@eyou.com (Yang); liforever7944@sina.com (Liu); 2 Mount Sinai School of Medicine, One Gustave L. Levy Place, New York, NY 10029, USA; E-mail: yongkui.jing@mssm.edu

**Keywords:** 5-Azacytidine analogues, antiproliferative activity, differentiation, leukemia, structure-activity relationship

## Abstract

Twenty-six 5-azacytidine analogues have been synthesized, including 4-amino-6-alkyl-1-pyranosyl/ribofuranosyl-1,3,5-triazin-2(1*H*)-ones **1a-j**, 6-amino-4-alkyl/aryl-1-pyranosyl/ribofuranosyl-1,3,5-triazin-2(1*H*)-ones **2a-f** and 4-amino-6-alkyl-1,3,5-triazin-2-yl-1-thio-pyranosides/ribofuranosides **3a-j**. The antiproliferative activities of these synthetic analogues were investigated in human leukemia HL-60 cells. Ribofuranosyl *S*-nucleoside **3a**, a bioisostere of 5-azacytidine, had a similar antiproliferative ability as that of the latter. Introduction of a methyl at the 6 position of 5-azacytidine and/or replacement of the ribofuranosyl moiety with pyranosyl sugars or disaccharides significantly decreased the antiproliferative activities of the 5-azacytidine derivatives. Several compounds with the replacement of pyranosyl sugars enhanced all-trans retinoic acid-induced differentiation ability in human leukemia HL-60 cells.

## Introduction

**Figure 1:**
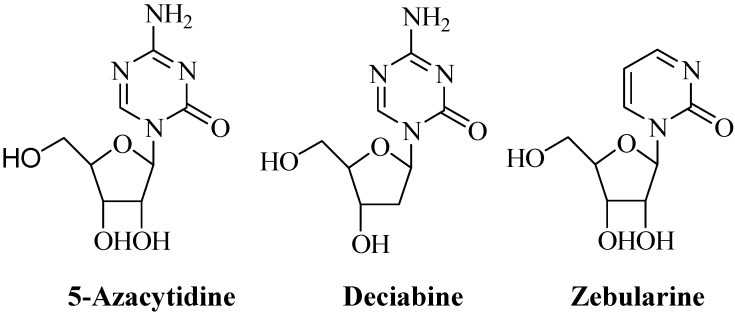
Structures of 5-Azacytidine, Decitabine and Zebularine

5-Azacytidine (5-aza-CR) and 5-aza-2’-deoxycytidine (5-aza-CdR, Decitabine) (Figure 1) are known DNA methyltransferase inhibitors and have been approved for the treatment of myelodysplastic syndrome (MDS) and chronic myelomonocytic leukemia (CMML) [1]. Although both agents are cytotoxic at high concentrations, the therapeutic effects of both compounds in MDS have been thought to be mediated through inhibition of the DNA methyltransferase at low concentrations [1]. Aberrant DNA methylation in the promoter region of genes can silence their expression [2]. Some tumor suppressor genes have been found to be silenced due to DNA hypermethylation and these genes can be reactivated by DNA demethylation [3]. Treatments of malignant cells with 5-aza-CR or 5-aza-CdR have been found to be associated with reversal of specific gene suppression [1,4]. Zebularine (Figure 1), a derivative of 5-aza-CR with the increased stability, has been reported to have little cytoxicity but to have maintained the ability of inhibiting DNA methytransferase activity [5,6]. Although 5-fluoro-deoxycytidine (FDAC) has both cytotoxic effects to malignant cells and inhibitory effects on DNA methyltransferase activity, 5-fluorouracil (5-FU) does not have the inhibitory effects on DNA methyltransferase activity [4]. These observations suggest that the riboside moiety of these nucleoside inhibitors is required for the inhibition of DNA methyltransferase activity [7]. Since the cytotoxic effects of these compounds are due to their incorporation into DNA or RNA [8,9], it seems that replacement of the riboside with other types of sugars will decrease the cytotoxic effects and will keep the abilities of DNA methyltransferase inhibition. 

We have synthesized a series of 5-aza-CR derivatives with introduction of a methyl or an ethyl group at the 6 position of 5-aza-CR, and/or with a replacement of O by S, or with a replacement of ribofuranosyl moiety by pyranosyl sugars or disaccharides. Addition of a methyl or an ethyl group at the 6 position of 5-aza-CR could block the attack of water and will improve the chemical stability of 5-aza-CR. The replacement of ribofuranosyl moiety with pyranosyl sugars or disaccharides is estimated to reduce cytotoxicity of 5-aza-CR. The antiproliferative activities as well as the differentiation induction of these derivatives alone and in combination with all-trans retinoic acid (ATRA) were investigated in human leukemia HL-60 cells.

## Results and Discussion

### Chemistry

**Scheme 1:**
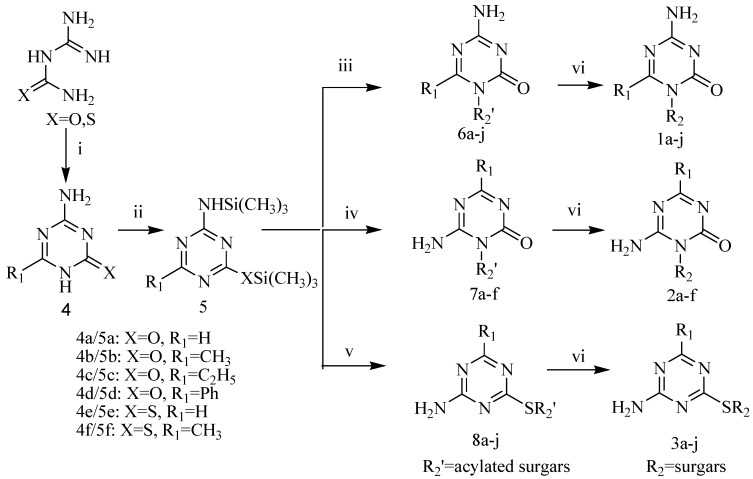
Synthetic route to the target compounds.

As illustrated in Scheme 1, the target compounds **1-3** were synthesized from guanylurea or guanylthiourea through the intermediate compounds **4-8**. 4-Amino-6-alkyl/aryl-1,3,5-triazin-2(1*H*)-ones **4a-f**, obtained in moderate yields from the reactions of guanylurea or guanylthiourea with orthoesters, reacted with hexamethyldisilazane (HMDS) to produce *N,O*-bistrimethylsilylated 1,3,5-triazinones **5a-f**. All reactions were carried out according to the procedures described in the literature [10,11,12,13,14]. Vorbrüggen coupling of **5a-f** and acylated sugars in anhydrous acetonitrile in the presence of a Lewis acid catalyst gave the acylated nucleosides in moderate yields. Purification of raw products by flash column chromatography gave acylated *N*_1_-nucleosides **6**, acylated *N*_3_-nucleosides **7** or acylated *S*-nucleosides **8** as main products. 4-Amino-6-alkyl-1-pyranosyl/ribofuranosyl-1,3,5-triazin-2(1*H*)-ones **1a-j**, 6-amino-4-alkyl/aryl-1-pyranosyl/ribofuranosyl-1,3,5-triazin-2(1*H*)-ones **2a-f** and 4-amino-6-alkyl-1,3,5-triazin-2-yl-1-thio-pyranosides/ribofuranosides **3a-j** were obtained after deprotection of acylated nucleosides **6-8** in a saturated methanol solution of ammonia at room temperature. The structures of target compounds are shown in Table 1.

*Reagents and conditions*: (i)orthoesters/DMF, 130 ^o^C, 80%-85%; (ii) HMDS/(NH_4_)_2_SO_4_, 92%-95%; (iii) acylated sugars/SnCl_4_ orTMSOTf/CH_3_CN, 40%-45%; (iv) Acylated ribose(pyranose)/SnCl_4_/ CH_3_CN, 24%-32%; (v) acylated ribose(pyranose)/TMSOTf/CH_3_CN, 30%-37%; vi: NH_3_/CH_3_OH, 50%-65%.

Vorbrüggen coupling of **5a** with acylated sugars and of **5b** with 1-*O*-acetyl-2,3,5-tri-*O*-benzoyl-β-d-ribose in anhydrous acetonitrile with SnCl_4_ or trimethylsilyltriflate (TMSOTf) [15] catalysis gave acylated nucleosides **6a-g**. However, when the coupling procedure was applied to **5b** and acetylated pyranoses in the presence of SnCl_4_ (2 equiv.), the acylated *N*_3_-isomers **7a-d** were obtained as the main products. The ratios of *N*_3_/*N*_1_ glucopyranoside, xylopyranoside, manopyranoside and maltopyranoside were 6.5:1, 5.5:1, 3.5:1, 8.2:1, respectively. It was observed that the *N*_1_-isomers **6h-j** were the predominant products when a weaker Lewis acid (TMSOTf, 2 equiv.) was used as the catalyst. The *N*_1_/*N*_3_ ratios of glucopyranoside, xylopyranoside and manopyranoside were 4:1, 5.2:1, 3.8:1, respectively. *N*_3_-Nucleosides **7e-f** were formed during couplings of **5c** and **5d** with 1-*O*-acetyl-2,3,5-tri-*O*-benzoyl-β-d-ribose under the same reaction conditions. The regioselectivity of nucleoside synthesis depended both on the substrates and catalysts. Basicity and steric hindrance of *N*_1_ in the *N,O*-bistrimethylsilylated 1,3,5-triazinones **5a-f** increased when a methyl, an ethyl or a phenyl was introduced to the 6 position. When the substitute was a methyl, a small electrophilic group, the basicity of *N*_1_ was the major factor affecting *N*_1_/*N*_3_ regioselectivity. Since TMSOTf is a weaker Lewis acid than SnCl_4_, less σ-complex was formed between TMSOTf and the *N*_1_ of **5b** and therefore more free *N*_1_ was present to form acylated *N*_1_-nucleosides **6h-j** [16]. When an ethyl or a phenyl was introduced to the 6 position the steric hindrance turned to be a major factor determining the regioselectivity. The *N*_3_-nucleosides **7e-f** were predominant products, even when TMSOTf was used as a catalyst. The acylated *S*-nucleosides **8a-j**, which are kinetically controlled products, were the predominant products during couplings of **5e** and **5f** with acylated sugars in anhydrous acetonitrile under SnCl_4_ or TMSOTf catalysis. Thus was presumably due to the stronger nucleophilicity of a sulphur compared to an oxygen.

The structures of all compounds were determined by application of IR, ^1^H-NMR and MS spectral data. The structure assignments for compound **1**, **2** and **3** were supported by comparing the ^1^H-NMR and HMBC spectral data with each other. The H-1’ peak was found to be correlated with C_2_ (C=O) and C_6_ (C-R_1_) in HMBC spectrum in *N*_1_-nucleosides **1**. There was a single peak for the two hydrogen signal of the amino group in ^1^H-NMR. However, the H-1’ peak was correlated with C_2_ (C=O) and C_6_ (C-NH_2_) in HMBC spectrum in *N*_3_-nucleosides **2**. There were two single peaks at about 8.31-8.46 and 6.94-7.87 ppm in ^1^H-NMR spectra corresponding to the two hydrogen signals of the amino group. This non-equivalence of the amino protons was caused by the pyranosyl sugar, which enhances the rotational barrier of the amino group. The *S*-nucleosides **3** were identified by HMBC spectra analysis. The H-1’ peak was found to be correlated with C_2_ (C-S) in HMBC spectrum. The H-1’ peak of *N*_3_-nucleosides was observed at about 5.85 ppm and these values slightly shifted to low field comparing to that of the *N*_1_-nucleosides and *S-*nucleosides (5.35 ppm). The configuration of the nucleosides was deduced from the trans-diaxial coupling between H-1’ and H-2’ of pyranose in the ^1^H-NMR spectra. **1c**, **1d**, **1j**, **2c**, **3d**, and **3i** were α-manopyranosyl or α-rhamnopyranosyl and β-configuration nucleosides.

### Bioactivity

The antiproliferative activities of these synthetic compounds were determined in human leukemia HL-60 cells. **5-aza-CR** is a potent growth inhibitor with a GI50 value of 0.29 μM. By using a trypan blue exclusion assay, we found that **5-aza-CR** killed half of cells at a concentration of 1.0 μM. Among all the listed compounds, only compound **3a** has a lower GI50 value of 1.7 μM. Compound **1g** has a GI50 value of 18.5 μM. The GI50 values of other compounds can not be obtained since these compounds do not inhibit 50% cell growth at concentrations less than 50 μM. Compound **3a** is the bioisostere of **5-aza-CR**, with a ribofuranosyl *S*-nucleoside. Compound **1g** only has an introduction of a methyl group at the 6 position of **5-aza-CR** without replacement of the ribofuranosyl group. Comparing the structures of **1a-f** with **5-aza-CR** it was found that replacement of the β-d-ribofuranosyl with a β-d-glucopyranosyl, a β-d-xylopyranosyl, a α-d-mannopyranosyl, a α-l-rhamnopyranosyl, a β-d-maltopyranosyl or a β-d-lactopyranosyl significantly decreased the antiproliferative activities. Comparison of the antiproliferative activities of **5-aza-CR** with compound **1g** revealed that replacement of the H with a CH_3_ (**1g**) slightly decreased the antiproliferative activities. However, replacement of the H (**3a**) with a CH_3_ (**3f**), where both are ribofuranosyl *S*-nucleosides, evidently decreased the antiproliferative activity. The antiproliferative activity of **5-aza-CR** is due to its incorporation into DNA and/or RNA [8], it seems that replacement of the β-d-ribofuranosyl in these nucleosides with other sugar moieties would not incorporate into DNA or RNA, that may explain their non-toxic effect to HL-60 cells. It has been shown that ATRA induced differentiation of human leukemia cells with induction of RARβ2 that has been found to be hypermethylated [17]. We have investigated the differentiation activities of these compounds alone and in combination with ATRA in HL-60 cells using NBT reduction assay as a differentiation marker. ATRA treatment at 0.1 μM induced 25.9% of HL-60 cells undergoing differentiation after 5 days. All of these compounds alone did not induce NBT reduction but some compounds such as **1c**, **1d** and **3i** enhanced ATRA-induced differentiation. The enhancement on ATRA differentiation induction by these compounds may be due to the enhanced RARβ2 expression through inhibition of DNA methyltransferase that needs to be further investigated. Since non-nucleoside compounds such as (-)-epigallocatechin-3-gallate, hydralazine and procainamide inhibit DNA methyltransferase activity [18,19], we prospect that some of these synthetic compounds will keep the ability of inhibiting DNA methyltransferase activity without incorporating into DNA. The inhibitory abilities of these compounds on DNA methyltransferase activity are under investigation. 

**Table 1:** Chemical structures, antiproliferative and differentiation induction abilities of target compounds in human leukemia HL-60 cells.

Compound	Structure		Growth Inhibition^a^ (%)	Differentiation^b^ Induction (%)
	R_1_	R_2_		
	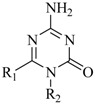			
**1a**	H	β-D-glucopyranosyl	14.4±8.4	21.2±1.2
1b*	H	β-D-xylopyranosyl	24.2±6.7	22.7±1.9
1c*	H	α-D-mannopyranosyl	24.0±5.2	34.8±3.7^#^
1d*	H	α-L-rhamnopyranosyl	24.5±12.0	34.4±1.7^##^
1e*	H	β-D-maltopyranosyl	36.1±7.3	25.0±1.2
1f*	H	β-D-lactopyranosyl	25.4±6.1	23.9±2.2
**1g**	CH_3_	β-D-ribofuranosyl	(18.5±3.9μM)	22.8±1.4(10μM)
1h*	CH_3_	β-D-glucopyranosyl	28.3±5.2	22.5±1.6
1i*	CH_3_	β-D-xylopyranosyl	40.7±9.9	21.3±1.1
1j*	CH_3_	α-D-mannopyranosyl	^ND^	^ND^
	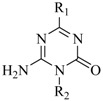			
2a*	CH_3_	β-D-glucopyranosyl	23.7±6.0	26.6±1.6
2b*	CH_3_	β-D-xylopyranosyl	22.0±5.3	22.6±2.1
2c*	CH_3_	α-D-mannopyranosyl	5.7±4.3	22.8±2.4
2d*	CH_3_	β-D-maltopyranosyl	25.9±8.8	20.8±1.5
2e*	C_2_H_5_	β-D-ribofuranosyl	3.3±1.8	24.8±2.4
**2f**	C_6_H_5_	β-D-ribofuranosyl	3.8±3.3	20.3±2.9
	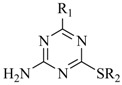			
3a*	H	β-D-ribofuranosyl	(1.7±0.1μM)	19.8±2.2(0.85μM)
3b*	H	β-D-glucopyranosyl	12.9±3.4	24.0±1.7
3c*	H	β-D-xylopyranosyl	32.1±9.3	21.3±1.5
3d*	H	α-D-mannopyranosyl	26.5±8.9	19.4±1.6
3e*	H	β-D-maltopyranosyl	2.1±2.1	23.5±1.9
3f*	CH_3_	β-D-ribofuranosyl	15.4±4.4	23.2±2.4
3g*	CH_3_	β-D-glucopyranosyl	2.6±2.6	20.6±1.9
3h*	CH_3_	β-D-xylopyranosyl	15.4±3.2	24.2±2.3
3i*	CH_3_	α-D-mannopyranosyl	36.0±11.9	31.4±2.3^#^
3j*	CH_3_	β-D-maltopyranosyl	20.0±5.1	21.7±1.3
**5-azaCR**			(0.29±0.015μM)	24.0±1.3(0.15μM)
**ATRA**				25.9±1.4

Notes: asterisked compounds are novel compounds. ND, not determined. ^a^^.^ Data shown are growth inhibition rates in HL-60 cells after treatment with the listed compounds at 50 μM for 72 h. The GI50 values of **1g**, **3a** and **5-aza-CR** are listed in parenthesis. Data shown are Mean±SD of three independent experiments. ^b^^.^ Data shown are percentage of NBT positive cells in HL-60 cells after treatment with the listed compounds at 50 μM or with **1g**, **3a** or **5-aza-CR** at the concentrations listed in parenthesis in combination with ATRA at 0.1μM for 120 h. Data shown are Mean ± SD of three independent experiments. # P<0.05; ## P<0.01 compared to cells treated with ATRA alone.

In summary, our data indicate that 1) changing **5-aza-CR** into a bioisosteric ribofuranosyl *S*-nucleoside (**3a**) does not influence the antiproliferative ability; 2) replacement of the ribofuranosyl moiety with pyranosyl sugars or disaccharides significantly decreases the antiproliferative efficacies; 3) compounds with the ribofuranosyl moiety replaced by the pyranosyl sugars (**1c**, **1d** and **3i**) enhance **ATRA** differentiation induction ability.

## Experimental

### General

The melting points were determined on an electrically heated X4 digital visual melting point apparatus and were uncorrected.. IR spectra were recorded on Bruker IFS 55 (KBr). ^1^H-NMR spectra were recorded on a Bruker ARX-300 spectrometer at 300MHz with DMSO-*d*_6_ as solvent and TMS as an internal standard. Mass spectra were taken in ESI mode on Agilent 1100 LC-MS. Elemental analysis was determined on a Carlo-Erba 1106 Elemental analysis instrument. All solvents and reagents were commercially available.

### General procedure for preparation target nucleosides

A mixture of guanylurea or guanylthiourea, orthoesters and dimethylformamide was refluxed for 90 min and allowed to stand at room temperature, the precipitate were recrystallized from the solution, then filtered off to give 4-amino-6-alkyl/aryl-1,3,5-triazin-2(1*H*)-ones **4a-f**. The structures were identified by MS [10,11,12]. Then a mixture of **4**, HMDS and (NH_4_)_2_SO_4_ or pyrimidine as catalyst was refluxed for 12-16 h. After evaporation under vacuum, the silylated bases **5a-f** were obtained [13,14]. The crude product was used in glycosylation without further purification. Silylated base (1 equiv) was dissolved in anhydrous acetonitrile, to this solution were added 1-*O*-acetyl-2,3,5-tri-*O*-benzoyl-β-d-ribose (1 equiv.) or acetylated pyranosyl sugar (1 equiv.) and SnCl_4_ (2 equiv.) or TMSOTf (2 equiv.) [15] in acetonitrile. The mixture was left standing at room temperature under stirring for 10-18 h, then diluted in chloroform and neutralized with a saturated solution of sodium bicarbonate. The organic layer was separated, dried and evaporated. The residue was purified by flash column chromatography (CHCl_3_-CH_3_OH). Acetylated nucleosides **6-8** were obtained as white solids which were identified by ^1^H-NMR and MS spectra. Then the acetylated nucleosides and saturated NH_3_-CH_3_OH solution were stirred at room temperature for 5 h and evaporated to dryness. The residue was purified by flash column chromatography (CHCl_3_-CH_3_OH), and recrystallized from water/methanol to give target nucleosides **1**-**3** as whte crystals. 

*4-Amino-1-β-d-glucopyranosyl-1,3,5-triazin-2(1H)-one* (**1a**). Yield: 21.6%; mp: 258-260°C (dec.) [20] (water/methanol); IR ν/cm^-1^: 3350.5, 1645.0, 1383.6, 1074.1, 902.4, 849.9, 796.5; ^1^H-NMR δ/ppm:3.18-3.19 (2H, m, H-6’, H-6”), 3.21-3.27 (2H, m, H-4’, H-5’), 3.56-3.57 (1H, m, H-3’),3.67-3.71 (1H, m, H-2’), 4.58 (1H, s, OH), 5.12 (1H, s, OH), 5.26 (1H, s, OH), 5.34 (1H, d, *J* = 9.4 Hz, H-1’), 5.38 (1H, s, OH), 7.59 (2H, s, NH_2_), 8.37 (1H, s, H-6). ESI-MS: 275.4[M+H]^+^; Anal. Calcd. for C_9_H_14_N_4_O_6_: C 39.42, H 5.15, N 20.43. Found: C 39.38, H 5.12, N 20.45.

*4-Amino-1-β-d-xylopyranosyl-1,3,5-triazin-2(1H)-one* (**1b**). Yield: 21.6%; mp: 191-193°C (water/ methanol); IR ν/cm^-1^: 3352.8, 1640.8, 1481.6, 1383.0, 1173.7, 1094.2, 1056.2, 897.9, 864.2, 797.8; ^1^H-NMR δ/ppm: 3.17-3.21 (3H, m, H-4’, H-5’, H-5”), 3.62-3.63 (1H, m, H-3’), 3.78-3.79 (1H, m, H-2’), 5.13 (1H, m, OH), 5.25-5.29 (2H, m, OH), 5.39 (1H, d, *J* = 4.2 Hz, H-1’), 7.61 (2H, s, NH_2_), 8.38 (1H, s, H-6). ESI-MS: 245.1 [M+H]^+^; Anal. Calcd. for C_8_H_12_N_4_O_5_: C 39.35, H 4.95, N 22.94. Found: C 39.42, H 4.90, N 22.98.

*4-Amino-1-α-d-mannopyranosyl-1,3,5-triazin-2(1H)-one* (**1c**). Yield: 16.8%; mp: 158-160°C (water/ methanol); IR ν/cm^-1^: 3396.0, 1695.1, 1383.4, 1098.7, 1051.4, 979.9; ^1^H-NMR δ/ppm: 3.60-3.64 (3H, m, H-4’, H-6’, H-6”), 3.79 (1H, s, H-5’), 3.87 (1H, s, H-3’), 4.11(1H, s, H-2’), 4.56 (1H, s, OH), 5.12-5.24 (3H, m, OH), 5.74-5.77 (1H, d, *J* = 9.0 Hz, H-1’), 7.55 (2H, s, NH_2_), 8.34 (1H, s, H-6); ESI-MS: 273.3 [M-H]^+^; Anal. Calcd. for C_9_H_14_N_4_O_6_: C 39.42, H 5.15, N 20.43. Found: C 39.44, H 5.08, N 20.39.

*4-Amino-1-(6'-deoxy-α-l-mannopyranosyl)-1,3,5-triazin-2(1H)-one* (**1d**). Yield: 12.4 %; mp: 126-128°C (water/methanol); IR ν/cm^-1^: 3391.4, 2922.3, 1639.8, 1511.5, 1383.5, 1054.1, 932.3, 798.4; ^1^H-NMR δ/ppm: 1.32-1.37 (3H, d, *J* = 6.9 Hz, CH_3_), 3.87 (2H, s, H-4’, H-3’), 3.95-3.98 (1H, d, *J* = 6.9 Hz, H-5’), 4.07 (1H, s, H-2’), 5.10-5.12 (1H, d, OH), 5.19 (2H, s, OH), 5.80-5.83 (1H, d, *J* = 9.6 Hz, H-1’), 7.50-7.53 (2H, d, NH_2_), 8.32 (1H, s, H-6); ESI-MS: 259.2 [M+H]^+^; Anal. Calcd. For C_9_H_14_N_4_O_5_: C 41.86, H 5.46, N 21.70. Found: C 41.92, H 5.49, N 21.76.

*4-Amino-1-(4'-O-α-d-glucopyranosyl-β-d-glucopyranosyl)-1,3,5-triazin-2(1H)-one* (**1e**). Yield: 19.6%; mp: 127-129 °C (water/methanol); IR ν/cm^-1^: 3369.2, 1697.4, 1382.4, 1169.6, 1088.4, 1038.6, 893.1, 780.2; ^1^H-NMR δ/ppm: 3.08-3.09 (1H, m), 3.25-3.26 (1H, m), 3.39-3.51 (5H, m), 3.54-3.59 (2H, m), 3.61-3.67 (2H, m), 3.70-3.72 (1H, m), 4.53-4.56 (2H, m, OH), 4.94 (2H, s, OH), 5.03-5.04 (1H, d, *J* = 3.4 Hz, H-1”), 5.39-5.41 (1H, d, *J* = 9.4 Hz, H-1’), 5.43-5.44 (1H, d, OH), 5.52 (1H, s, OH), 5.71 (1H, s, OH), 7.58-7.60 (2H, d, NH_2_), 8.39 (1H, s, H-6); ESI-MS: 437.4 [M+H]^+^; Anal. Calcd. for C_15_H_24_N_4_O_11_: C 41.29, H 5.54, N 12.84. Found C 41.36, H 5.58, N 12.80.

*4-Amino-1-(4'-O-β-d-galactopyranosyl-β-d-glucopyranosyl)-1,3,5-triazin-2(1H)-one* (**1f**). Yield: 20.8%; mp: 181-182 °C (water/methanol); IR ν/cm^-1^: 3385.3, 1689.5, 1085.2, 896.3, 782.6; ^1^H-NMR δ/ppm: 3.35-3.60 (7H, m), 3.63-3.72 (3H, m), 3.75-3.76 (2H, m), 4.22 (1H, s, OH), 4.56-4.60 (2H, d, OH), 4.69 (1H, s, OH), 4.82 (2H, s, H-1”, OH), 5.15(1H, s, OH), 5.41-5.44 (2H, d, *J* = 9.2 Hz, H-1’), 5.46 (1H, s, OH), 7.59 (2H, s, NH_2_), 8.38(1H, s, H-6); ESI-MS: 437.3 [M+H]^+^; Anal. Calcd. For C_15_H_24_N_4_O_11_: C 41.29, H 5.54, N 12.84. Found: C 41.32, H 5.62, N 12.86.

*4-Amino-6-methyl-1-β-d-ribofuranosyl-1,3,5-triazin-2(1H)-one* (**1g**). Yield: 23.5%; mp: 142-145 °C [13] (water/methanol); IR ν/cm^-1^: 3359.6, 1650.8, 1594.7, 1105.4, 895.1, 797.8; ^1^H-NMR δ/ppm: 2.43 (3H, s, CH_3_), 3.45-3.47 (2H, m, H-5’, H-5”), 3.59-3.63 (1H, m, H-4’), 3.75-3.76 (1H, d, H-3’), 4.56 (1H, s, H-2’), 4.75 (1H, s, OH), 4.97 (1H, s, OH), 5.20 (1H, s, OH), 5.59-5.61 (1H, d, H-1’), 7.46-7.47 (2H, s, NH_2_); ESI-MS: 259.2 [M+H]^+^; Anal. Calcd. for C_9_H_14_N_4_O_5_: C 41.86, H 5.46, N 21.70. Found C 41.89, H 5.43, N 21.75.

*4-Amino-β-d-glucopyranosyl-6-methyl-1,3,5-triazin-2(1H)-one* (**1h**). Yield: 16.8%; mp: 168-169 °C (water/methanol); IR ν/cm^-1^: 3373.2, 2926.8, 1636.9, 1526.2, 1383.8, 1190.4, 1077.8, 893.3, 797.7; ^1^H-NMR δ/ppm: 2.44( 3H, m, CH_3_), 3.22-3.25 (2H, m, H-6’, H-6”), 3.45-3.47 (2H, m, H-4’, H-5’), 3.67-3.71 (2H, d, H-3’), 4.21-4.52 (5H, br, OH, H-2’), 5.26-5.27 (1H, d, *J* = 5.2 Hz, H-1’); 7.32 (2H, s, NH_2_); ESI-MS: 287.4 [M-H]^-^; Anal. Calcd. for C_10_H_16_N_4_O_6_: C 41.67, H 5.59, N 19.44. Found C 41.56, H 5.64, N 19.49.

*4-Amino-6-methyl-1-β-d-xylopyranosyl-1,3,5-triazin-2(1H)-one* (**1i**). Yyield 19.7%; mp: 156-158 °C (water/methanol); IR ν/cm^-1^: 3406.9, 2923.4, 1669.1, 1301.2, 1048.0, 978.0, 943.5, 896.2; ^1^H-NMR δ/ppm: 2.41 (3H, s, CH_3_), 3.16-3.20 (4H, m, H-3’, H-4’, H-5’, H-5”), 3.81-3.82 (1H, m，H-2’), 5.08-5.13 (4H, m, br, OH), 5.35-5.36 (1H, d, *J* = 8.5 Hz, H-1’), 7.30 (2H, s, NH_2_); ESI-MS: 257.3 [M-H]^+^; Anal. Calcd. for C_9_H_14_N_4_O_5_: C 41.86, H 5.46, N 21.70. Found C 41.80, H5.38, N 21.72.

*4-Amino-1-α-d-mannopyranosyl-6-methyl-1,3,5-triazin-2(1H)-one* (**1j**). Yield: 14.7 %; mp: 173-175 °C (water/methanol); IR ν/cm^-1^: 3383.8, 2926.3, 1633.7, 1529.3, 1384.2, 1113.7, 1055.0, 922.2, 865.0, 797.9; ^1^H-NMR δ/ppm: 2.47 (3H, s, CH_3_), 3.56 (1H, m, H-6”), 3.63-3.70 (2H, m, H-4’, H-5’), 3.84-3.85 (1H, m, H-3’), 3.91-3.94 (1H, m, H-6’), 4.52-4.56 (2H, m, OH, H-2’), 4.95-4.97 (1H, d, OH), 5.04-5.08 (2H, OH), 5.93-5.96 (1H, d, *J* = 8.9 Hz, H-1’), 7.25 (2H, s, NH_2_); ESI-MS: 287.2 [M-H]^-^; Anal. Calcd. for C_10_H_16_N_4_O_6_: C 41.67, H 5.59, N 19.44. Found C 41.58, H 5.54, N 19.35.

*6-Amino-1-β-d-glucopyranosyl-4-methyl-1,3,5-triazin-2(1H)-one* (**2a**). Yield: 10.6%; mp: 181-182 °C (water/methanol); IR ν/cm^-1^: 3394.1, 1688.5, 1564.4, 1069.4, 899.9, 795.4; ^1^H-NMR δ/ppm: 2.06 (3H,s,CH_3_), 3.23-3.36 (2H, m, H-6’, H-6”), 3.49-3.62 (3H, m, H-3’, H-4’, H-5’), 3.73 (1H, s, H-2’), 4.73-4.77 (1H, t, OH), 5.16-5.18 (1H, d, OH), 5.25-5.26 (1H, d, OH), 5.40-5.42 (1H, d, OH), 5.80 (1H, s, H-1’), 6.94 (1H, s, NH), 8.35 (1H, s, NH); ESI-MS: 287.3 [M-H]^+^; Anal. Calcd. for C_10_H_16_N_4_O_6_: C 41.67, H 5.59, N 19.44. Found C 41.58, H 5.57, N 19.38. 

*6-Amino-4-methyl-1-β-d-xylopyranosyl-1,3,5-triazin-2(1H)-one* (**2b**). Yield: 12.1%; mp: 170-172 °C (water/methanol); IR ν/cm^-1^: 3378.7, 1692.2, 1565.2, 1384.1, 1091.9, 1038.3, 895.4, 846.6, 797.5; ^1^H-NMR δ/ppm: 2.08 (3H, s, CH_3_), 3.18 (2H, s, H-5’, H-5”), 3.66-3.83 (3H, m, H-2’, H-3’, H-4’), 5.04-5.06 (1H, d, OH), 5.19(1H, s, OH), 5.36-5.38 (1H, d, OH), 5.75(1H, s, br, H-1’), 7.04 (1H, s, br, NH), 8.31 (1H, s, br, NH); ESI-MS: 258.7 [M+H]^+^; Anal. Calcd. for C_9_H_14_N_4_O_5_: C 41.86, H 5.46, N 21.70. Found C 41.80, H 5.40, N 21.63.

*6-Amino-1-α-d-mannopyranosyl-4-methyl-1,3,5-triazin-2(1H)-one* (**2c**). Yield: 8.9%; mp: 162-164 °C (water/methanol); IR ν/cm^-1^: 3400.5, 1687.0, 1558.7, 1481.7, 1384.6, 1050.0, 1012.3, 921.4, 866.8, 796.4; ^1^H-NMR δ/ppm: 2.09 (3H, s, CH_3_), 3.54-3.57 (1H, m, H-6”), 3.73 (1H, s, H-3’), 3.89 (3H, s, H-4’, H-5’, H-6’), 4.28-4.30 (1H, m, H-2’), 4.73 (1H, s, OH), 5.05-5.07 (1H, d, OH), 5.21-5.22 (1H, d, OH), 5.51 (1H, s, OH), 6.27-6.30 (1H, d, *J* = 9.7 Hz, H-1’), 6.98 (1H, s, br, NH), 8.46 (1H, s, br, NH); ESI-MS: 287 [M-H]^+^; Anal. Calcd. for C_10_H_16_N_4_O_6_: C 41.67, H 5.59, N 19.44. Found C 41.56, H 5.64, N 19.49.

*6-Amino-1-(4'-O-α-d-glucopyranosyl-β-d-glucopyranosyl)-4-methyl-1,3,5-triazin-2(1H)-one* (**2d**). Yield: 14.6%; mp: 175-177 °C (water/methanol); IR ν/cm^-1^: 3418.6, 2923.2, 1692.2, 1379.9, 1059.2, 918.6, 893.2, 869.0, 840.9, 798.3; ^1^H-NMR δ/ppm: 2.11 (3H, s, CH_3_), 3.10-3.12 (1H, m), 3.28 (2H, s), 3.60-3.83 (8H, m), 4.47 (1H, s, OH), 4.56 (1H, s, OH), 4.78 (1H, s, OH), 4.97 (2H, s, OH, H-2’), 5.02-5.03 (1H, s, H-1”), 5.54 (1H, s, OH), 5.58-5.60 (1H, d, OH), 5.74 (1H, s, OH), 5.89 (1H, s, H-1’), 7.02 (1H, s, br, NH), 8.40 (1H, s, br, NH); ESI-MS: 449.4 [M-H]^+^; Anal. Calcd. for C_16_H_26_N_4_O_11_: C 42.67, H 5.82, N 12.44. Found C 42.55, H 5.93, N 12.49.

*6-Amino-4-ethyl-1-β-d-ribofuranosyl-1,3,5-triazin-2(1H)-one* (**2e**). Yield: 10.3%; mp: 180-182 °C (water/methanol); IR ν/cm^-1^: 3314.9, 1682.0, 1655.5, 1552.7, 1115.2, 1085.0, 900.3, 876.1, 854.0, 806.1; ^1^H-NMR δ/ppm: 1.11-1.16 (3H, t, *J* = 7.5 Hz, CH_3_), 2.32-2.40 (2H, q, *J* = 7.5 Hz, CH_2_), 3.57-3.67 (2H, m, H-5’, H-5”), 3.94 (1H, s, H-3’), 4.03 (1H, s, H-2’), 4.34-4.41 (1H, m, H-4’), 5.08-5.09 (1H, d, OH), 5.24-5.26 (1H, d, OH), 5.72 (1H, s, H-1’), 6.31-6.34 (1H, d, OH), 7.87(1H, s, br, NH), 8.38 (1H, s, br, NH); ESI-MS: 273.2 [M+H]^+^; Anal. Calcd. for C_10_H_16_N_4_O_5_: C 44.12, H 5.92, N 20.58. Found C 44.32, H 5.96, N 20.54.

*6-Amino-4-phenyl-1-β-d-ribofuranosyl-1,3,5-triazin-2(1H)-one* (**2f**). Yield: 17.8%; mp: 200-202 °C (dec.) [12] (water/methanol); IR ν/cm^-1^: 3383.5, 1680.8, 1478.4, 1109.5, 1077.4, 902.9, 786.0; ^1^H-NMR δ/ppm: 3.63-3.69 (2H, m, H-5’, H-5”), 3.97 (1H, s, H-4’), 4.06 (1H, s, H-3’), 4.42-4.42-4.45 (1H, m, H-2’), 5.14-5.15 (1H, d, OH), 5.33-5.36 (1H, d, OH), 5.81 (1H, s, OH), 6.39-6.41 (1H, d, OH), 7.47-7.57 (3H, m, aromatic H ), 8.07 (1H, s, br, NH), 8.25-8.31(2H, m, aromatic H), 8.59 (1H, s, br, NH); ESI-MS: 321.1[M+H]^+^; Anal. Calcd. for C_14_H_16_N_4_O_5_: C 52.50, H 5.03, N 17.49. Found C 52.48, H 5.08, N 17.52.

*4-Amino-1,3,5-triazin-2-yl-1-thio-β-d-ribofuranoside* (**3a**). Yield: 15.9%; mp: 117-118 °C (water/ methanol); IR ν/cm^-1^: 3278.4, 1682.1, 1569.6, 1212.3, 1066.0, 1047.9, 985.6, 935.3, 915.7, 831.7, 756.7; ^1^H-NMR δ/ppm: 3.40-3.45 (2H, m, H-5’, H-5”), 3.78-3.79 (1H, d, H-4’), 3.90-3.92 (1H, d, H-3’), 4.00-4.02 (1H, d, *J* = 4.5 Hz, H-2’), 4.78 (1H, s, OH), 5.01-5.02 (1H, d, OH), 5.38-5.40 (1H, d, OH), 5.86-5.87 (1H, d, *J* = 4.5 Hz, H-1’), 7.62 (2H, s, NH_2_), 8.24 (1H, s, H-6); ESI-MS: 261.1 [M+H]^+^; Anal. Calcd. for C_8_H_12_N_4_O_4_S: C 36.92, H 4.65, N 21.53. Found C 36.81, H 4.69, N 21.59.

*4-Amino-1,3,5-triazin-2-yl-1-thio-β-d-glucopyranoside* (**3b**). Yield: 10.7%; mp: 134-135 °C (water/ methanol); IR ν/cm^-1^: 3334.0, 2918.7, 1647.5, 1564.8, 1384.8, 1195.1, 1044.3, 942.2, 877.3, 808.4, 759.3; ^1^H-NMR δ/ppm: 3.16 (4H, s, H-4’, H-5’, H-6’, H-6”), 3.43-3.46(1H, m, H-2’), 3.61-3.62 (1H, m, H-3’), 4.48 (1H, s, OH), 5.00 (1H, s, OH), 5.14-5.15 (1H, d, OH), 5.34-5.38 (1H,d, *J* = 9.9 Hz, H-1’), 5.40-5.42 (1H, d, OH), 7.56-7.59 (2H, d, NH_2_), 8.25 (1H, s, H-6); ESI-MS: 291.3 [M+H]^+^; Anal. Calcd. for C_9_H_14_N_4_O_5_S: C 37.24, H 4.86, N 19.30. Found C 37.34, H 4.89, N 19.38.

*4-Amino-1,3,5-triazin-2-yl-1-thio-β-d-xylopyranoside* (**3c**). Yield: 14.6%; mp: 150-152 °C (water/ methanol); IR ν/cm^-1^: 3288.0, 1691.9, 1575.4, 1382.2, 110.7, 1068.6, 900.9, 833.4, 804.1, 757.7; ^1^H-NMR δ/ppm: 3.12-3.23 (4H,m, H-2’, H-3’, H-4’, H-5”), 3.77-3.83 (1H, m, H-5’), 5.06-5.08 (1H, d, OH), 5.22-5.24 (1H, d, OH), 5.38-5.41 (1H, d, *J* = 8.7 Hz, H-1’), 5.42-5.44 (1H, OH), 7.59 (2H, s, NH_2_), 8.25(1H, s, H-6); ESI-MS: 259.2 [M-H]^+^; Anal. Calcd. for C_8_H_12_N_4_O_4_S: C 36.92, H 4.65, N 21.53. Found C 36.98, H 4.58, N 21.50.

*4-Amino-1,3,5-triazin-2-yl-1-thio-α-d-mannopyranoside* (**3d**). Yield: 11.7%; mp: 116-118 °C (water/ methanol); IR ν/cm^-1^: 3401.9, 1652.3, 1564.4, 1384.1, 1105.1, 880.7, 753.7; ^1^H-NMR δ/ppm: 3.17-3.44 (4H, m, H-4’, H-5’, H-6’, H-6”), 3.62 (1H, s, H-3’), 3.84 (1H, s, H-2’), 4.50 (1H, s, OH), 4.89 (2H, s, OH), 5.24 (1H, s, OH), 6.30 (1H, s, H-1’), 7.66 (2H, s, NH_2_), 8.27 (1H, s, H-6); ESI-MS: 291.1 [M+H]^+^; Anal. Calcd. for C_9_H_14_N_4_O_5_S: C 37.24, H 4.86, N 19.30. Found C 37.16, H 4.85, N 19.37.

*4-Amino-4’-O-α-d-glucopyranosyl-1,3,5-triazin-2-yl-1-thio-β-d-glucopyranoside* (**3e**). Yield: 14.3%; mp: 191-193 °C (water/methanol); IR ν/cm^-1^: 3404.3, 2923.2, 1642.0, 1566.0, 1518.8, 1384.2, 1032.7, 810.5, 759.3; ^1^H-NMR δ/ppm: 3.06-3.31 (3H, m), 3.40-3.46 (6H, m), 3.59-3.62 (3H, m, H-2’, H-2”, H-3’), 4.48-4.50 (2H, m, OH), 4.88-4.93 (2H, OH), 5.03-5.04 (1H, d, *J* = 3.6 Hz, H-1”), 5.36-5.40 (1H, d, *J* = 10.5 Hz, H-1’), 5.42-5.44 (1H, d, OH), 5.51-5.53 (1H, d, OH), 5.67-5.68 (1H, d, OH), 7.55-7.61 (2H, d, NH_2_), 8.25 (1H, s, H-6); ESI-MS: 453.2 [M+H]^+^; Anal. Calcd. for C_15_H_24_N_4_O_10_S: C 39.82, H 5.35, N 12.38. Found C 39.76, H 5.28, N 12.34.

*4-Amino-6-methyl-1,3,5-triazin-2-yl-1-thio-β-d-ribofuranoside* (**3f**). Yield: 16.8%; mp: 117-118 °C (water/methanol); IR ν/cm^-1^: 3406.9, 3180.9, 1662.1, 1557.3, 1043.3, 801.9; ^1^H-NMR δ/ppm: 2.20 (3H, s, CH_3_), 3.39-3.41 (1H, m, H-5”), 3.45-3.46 (1H, m, H-5’), 3.78-3.79 (1H, m, H-4’), 3.89-3.91 (1H, m, H-3’), 3.98-4.01 (1H, m, H-2’), 4.76-4.78 (1H, m, OH), 5.01-5.03 (1H, d, OH), 5.38-5.40 (1H, d, OH), 5.88-5.89 (1H, d, *J* = 4.5 Hz, H-1’), 7.47 (2H, s, NH_2_); ESI-MS: 275.1 [M+H]^+^; Anal Calcd. for C_9_H_14_N_4_O_4_S: C 39.41, H 5.14, N 20.43. Found C 39.52, H 5.18, N 20.45.

*4-Amino-6-methyl-1,3,5-triazin-2-yl-1-thio-β-d-glucopyranoside* (**3g**). Yield: 15.9%; mp: 117-119 °C (water/methanol); IR ν/cm^-1^: 3340.1, 1644.8, 1545.3, 1384.3, 1283.5, 1049.1, 879.0, 820.7; ^1^H-NMR δ/ppm: 2.21 (3H, s, CH_3_), 3.12-3.17 (3H, m, H-5’, H-6’, H-6”), 3.21-3.22 (1H, m, H-4’), 3.44-3.45 (1H, m, H-2’), 3.60-3.61 (1H, m, H-3’), 4.46-4.48 (1H, t, OH), 5.00-5.01 (1H, d, OH), 5.14-5.15 (1H, d, OH), 5.37-5.38 (1H, d, *J* = 10.4 Hz, H-1’), 5.39-5.40 (1H, d, OH), 7.40 (1H, s, NH), 7.45 (1H, s, NH); ESI-MS: 303.3 [M-H]^+^; Anal. Calcd. for C_10_H_16_N_4_O_5_S: C 39.47, H 5.30, N 18.41. Found C 39.53, H 5.32, N 18.52.

*4-Amino-6-methyl-1,3,5-triazin-2-yl-1-thio-β-d-xylopyranoside* (**3h**). White crystal; yield: 13.8%; mp: 150-152 °C (water/methanol); IR ν/cm^-1^: 3280.5, 1684.9, 1582.4, 1379.2, 1068.6, 902.5, 836.6, 759.2; ^1^H-NMR δ/ppm: 2.22 (3H, s, CH_3_), 3.12-3.23 (3H, m, H-3’, H-4’, H-5”), 3.39 (1H, m, H-2’), 3.78-3.82 (1H, m, H-5’), 5.05-5.07 (1H, d, OH), 5.21-5.22 (1H, s, OH), 5.40-5.44 (2H, m, OH, H-1’), 7.45 (2H, d, NH_2_). ESI-MS: 273.4 [M-H]^+^; Anal. Calcd. for C_9_H_14_N_4_O_4_S: C 39.41, H 5.14, N 20.43. Found C 39.50, H 5.32, N 20.46.

*4-Amino-6-methyl-1,3,5-triazin-2-yl-1-thio-α-d-mannopyranoside* (**3i**). Yield: 12.4%; mp: 199-201 °C (water/methanol); IR ν/cm^-1^: 3339.3, 1654.6, 1553.5, 1281.2, 1212.6, 1071.6, 973.5, 899.8, 844.5, 804.3, 766.0; ^1^H-NMR δ/ppm: 2.23 (3H, s, CH_3_), 3.44-3.48 (4H, m, H-3’, H-4’, H-5’, H-6’), 3.61-3.65 (1H, d, H-6”), 3.83 (1H, s, H-2’), 4.49 (1H, s, OH), 4.88 (2H, s, OH), 5.21 (1H, s, OH), 6.34 (1H, s, H-1’), 7.49-7.51 (2H, d, NH_2_); ESI-MS: 303.4 [M-H]^+^; Anal. Calcd. for C_10_H_16_N_4_O_5_S: C 39.47, H 5.30, N 18.41. Found C 39.52, H 5.36, N 18.44.

*4-Amino-4’-O-α-d-glucopyranosyl-6-methyl-1,3,5-triazin-2-yl-1-thio-β-d-glucopyranoside* (**3j**). Yield: 16.8%; mp: 194-196 °C (water/methanol); IR ν/cm^-1^: 3384.5, 1645.0, 1556.8, 1281.5, 1074.0, 895.2, 805.0; ^1^H-NMR δ/ppm: 2.24 (3H, s, CH_3_), 3.23-3.26 (2H, m), 3.34-3.52 (2H, m), 3.54-3.55 (3H, m), 3.56-3.58 (5H, m), 3.65-3.72 (3H, m), 4.24-4.27 (1H, d, OH), 4.28-5.32 (5H, br, OH), 5.41-5.45 (1H, d, *J* = 10.3 Hz, H-1’), 7.44-7.49 (2H, d, NH_2_); ESI-MS: 465.4 [M-H]^+^; Anal. Calcd. for C_16_H_26_N_4_O_10_S: C 41.20, H 5.62, N 12.01. Found C 41.32, H 5.68, N 12.05.

### Biological activity assay

### Cell culture

Human leukemia HL-60 cells obtained from ATCC were cultured in RPMI 1640. The media were supplemented with 100 units/mL penicillin, 100 μg/mL streptomycin, 0.2％ NaHCO_3_, and 10% (v/v) heat-inactivated fetal bovine serum (FBS). 

### Cell growth inhibition

Cells were seeded at 5 × 10^4^ cells/mL and incubated with various concentrations of the indicated agents for three days. The total cell number in each group was determined with the aid of a hemocytometer and the drug concentration that inhibits half of cell growth (GI50) was calculated. The cell viability was estimated by trypan-blue exclusion assay.

### NBT reduction assay

The nitroblue tetrazolium (NBT) reduction assay as a determination of cell differentiation was performed as reported previously [21] and the cells were treated with 0.15 μM **5-aza-CR**, 0.85 μM **3a**, 10 μM **1g** or 50 μM each of other compounds alone and in combination with 0.1 μM **ATRA** for 5 days. Total 300 cells were counted and percentages of NBT positive cells were calculated. 
